# Modified versus standard intention-to-treat reporting: Are there differences in methodological quality, sponsorship, and findings in randomized trials? A cross-sectional study

**DOI:** 10.1186/1745-6215-12-58

**Published:** 2011-02-28

**Authors:** Alessandro Montedori, Maria Isabella Bonacini, Giovanni Casazza, Maria Laura Luchetta, Piergiorgio Duca, Francesco Cozzolino, Iosief Abraha

**Affiliations:** 1 Regional Health Authority of Umbria, Perugia, Italy; 2 Pharmacy Department, Derriford Hospital, NHS Trust, Plymouth, UK; 3 Istituto di Statistica Medica e Biometria, Università degli Studi di Milano, Milan, Italy; 4 Azienda Sanitaria Locale 3, Foligno, Italy; 5 Dipartimento di Scienze Cliniche, Università degli Studi di Milano, Milan, Italy

## Abstract

**Background:**

Randomized controlled trials (RCTs) that use the modified intention-to-treat (mITT) approach are increasingly being published. Such trials have a preponderance of post-randomization exclusions, industry sponsorship, and favourable findings, and little is known whether in terms of these items mITT trials are different with respect to trials that report a standard intention-to-treat.

**Methods:**

To determine differences in the methodological quality, sponsorship, authors' conflicts of interest, and findings among trials with different "types" of intention-to-treat, we undertook a cross-sectional study of RCTs published in 2006 in three general medical journals (the Journal of the American Medical Association, the New England Journal of Medicine and the Lancet) and three specialty journals (Antimicrobial Agents and Chemotherapy, the American Heart Journal and the Journal of Clinical Oncology). Trials were categorized based on the "type" of intention-to-treat reporting as follows: ITT, trials reporting the use of standard ITT approach; mITT, trials reporting the use of a "modified intention-to-treat" approach; and "no ITT", trials not reporting the use of any intention-to-treat approach. Two pairs of reviewers independently extracted the data in duplicate. The strength of the associations between the "type" of intention-to-treat reporting and the quality of reporting (sample size calculation, flow-chart, lost to follow-up), the methodological quality of the trials (sequence generation, allocation concealment, and blinding), the funding source, and the findings was determined. Odds ratios (OR) were calculated with 95% confidence intervals (CI).

**Results:**

Of the 367 RCTs included, 197 were classified as ITT, 56 as mITT, and 114 as "no ITT" trials. The quality of reporting and the methodological quality of the mITT trials were similar to those of the ITT trials; however, the mITT trials were more likely to report post-randomization exclusions (adjusted OR 3.43 [95%CI, 1.70 to 6.95]; *P *< 0.001). We found a strong association between trials classified as mITT and for-profit agency sponsorship (adjusted OR 7.41 [95%CI, 3.14 to 17.48]; *P *< .001) as well as the presence of authors' conflicts of interest (adjusted OR 5.14 [95%CI, 2.12 to 12.48]; *P *< .001). There was no association between mITT reporting and favourable results; in general, however, trials with for-profit agency sponsorship were significantly associated with favourable results (adjusted OR 2.30; [95%CI, 1.28 to 4.16]; *P *= 0.006).

**Conclusion:**

We found that the mITT trials were significantly more likely to perform post-randomization exclusions and were strongly associated with industry funding and authors' conflicts of interest.

## Background

The intention-to-treat principle requires that all participants that are randomized must be included in the final analysis and analyzed according to the treatment group to which they were originally assigned, regardless of the treatment received, withdrawals, lost to follow-up or cross-overs. Despite this principle, in many instances in randomized trials the term intention-to-treat was inappropriately described and participants improperly excluded[1-4]. In addition, the use of a modified intention-to-treat (mITT) approach in randomized controlled trials (RCTs) is increasingly appearing in the medical literature according to a systematic review[5]. There is no clear definition of what is mITT. In fact, descriptions of mITT analyses vary greatly from trial to trial, and often contain more than one criterion that are difficult to interpret as it is not easy to discriminate between missing data cases and deviations from protocol. Moreover, while post-randomization exclusion appear to be the primary factor that characterizes the mITT analysis, the majority of the trials were industry sponsored and reported results that favoured the therapy under investigation[5]. The uncertainty or equipoise principle that should exist in the design of RCTs states that over time, the mean benefit of investigational therapies and comparison therapies should be equal [6]. The high prevalence of significant results reported in randomized trials might not reflect true differences[7]. Some argue that this principle can be violated in the presence of financial ties between investigators and the pharmaceutical industry [8]. The biomedical literature reports strong and consistent evidence that industry-sponsored research is likely to produce pro-industry conclusions [9]. The appearance of mITT reporting in modern RCTs could be a consequence of the widespread financial ties that exists among investigators and industry.

In this cross-sectional study, we examined whether mITT reporting trials were different in terms of methodological quality from trials with other types of intention-to-treat reporting. Our first hypothesis was that studies using mITT analyses would be associated with limited quality of reporting and study characteristics based on the assumption that mITT reporting is a limitation in principle. Moreover, we investigated whether the reporting of mITT in RCTs was associated with industry sponsorship, the presence of authors' conflicts of interest, and favourable findings.

## Methods

### Selection of Studies

To compare methodological quality, industry sponsorship, the presence of authors' conflicts of interest, and findings among trials based on the type of intention-to-treat reporting, we sought to identify the top medical journals and specialty journals published in 2006 that were more likely to report the use of an mITT approach according to our previous survey [10]. The three high-ranking medical journals were the Journal of American Medical Association, the New England Journal of Medicine and the Lancet; and the three specialty journals were Antimicrobial Agents and Chemotherapy, the American Heart Journal and the Journal of Clinical Oncology. We carried out a computerized search in Medline (via PubMed) to identify RCTs published in these six journals using the publication type "randomized controlled trials". The results were then cross-checked against a search of the Cochrane Central Register of Controlled Trials.

**Figure 1:**
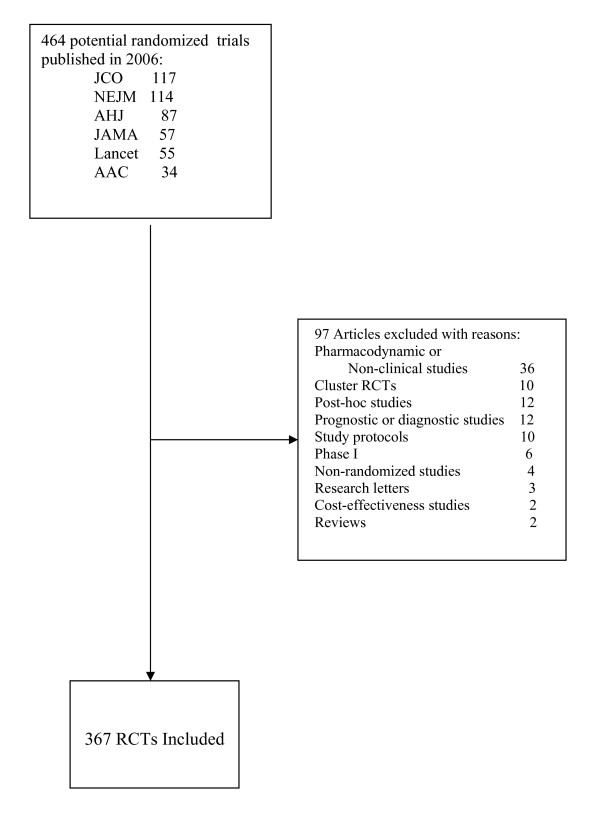
**Study Screening Process**. AAC: Antimicrobial Agents and Chemotherapy; AHJ: American Heart Journal; JAMA: Journal of American Medical Association; JCO: Journal of Clinical Oncology; NEJM: New England Journal of Medicine.

Excluded reports included: reports of phase I trials; pharmacokinetic, pharmacodymanic or dose-comparing studies; cluster RCTs; post-hoc studies; research letters; cost-effectiveness studies; study protocols; and prognostic and diagnostic studies. Two authors screened all of the full-text journal articles to confirm their eligibility for inclusion and excluded 97 articles (Figure 1).

Two other reviewers independently and in duplicate, extracted the following information from the journal articles: characteristics of the trial, primary outcomes, number of allocation groups, number of patients, main outcome measure, *P*-values, and number of subjects excluded from the analysis. Moreover, reporting of flow-charts, sample size calculations, and information regarding missing data, withdrawals or patients lost to follow-up were recorded.

The relevant RCTs were subsequently classified according to the type of intention-to-treat analyses used as follows: ITT, trials reporting the use of standard ITT analyses; mITT, trials reporting the use of "modified intention-to-treat" analyses; or "no ITT" trials not reporting the use of any intention-to-treat analyses. This classification was independent of the reporting of any post-randomisation exclusion. Trials reporting the use of ITT with descriptions or conditions different from the standard intention-to-treat definition were classified as mITT.

Descriptions of mITT were retrieved to evaluate the type and deviation from a true intention-to-treat analysis according to our previous classification[5].

### Assessment of Methodological Quality

Sequence generation, allocation concealment and blinding were used as indicators of methodological quality and were classified as "adequate," "inadequate" or "unclear." In our analysis, the "inadequate" and "unclear" categories were collapsed into "not adequate." For sequence generation, the following approaches were considered adequate: random number table, computer random number generator, drawing of lots and envelopes. The use of methods such as case record number, date of birth or day, month, or year of admission were considered inadequate for sequence generation.

For allocation concealment, central randomisation, coded drug packs prepared by an independent pharmacist and sequentially numbered, sealed and opaque envelopes or any method that hindered the researchers' ability to foresee the next assignment was considered adequate. Methods such as procedures based on inadequate generation of allocation sequences, open allocation schedules, alternation and unsealed or non-opaque envelopes were considered inadequate.

Regarding blinding, a trial was considered adequately blinded if it was described as "double-blinded" and used adequate methods such as identical placebo tablets or adequate descriptions of who was blinded (e.g., the outcome assessor) in cases where blinding of participants and caregivers may not be feasible. Each eligible trial was independently reviewed for methodological quality. Discrepancies were resolved by discussion, and a consensus was reached for all trials.

### Funding Source

Information regarding the sources of funding for each trial was extracted from the articles by reviewing the article text, the conflict of interest section, information on funding (if present) and the acknowledgements section. RTC funding sources were abstracted and categorized as follows: (1) not-for-profit organization; (2) for-profit agency; (3) co-financed, indicating funding from both not-for-profit organization(s) and for-profit agency(s); (4) no funding; and (5) not reported.

### Authors' Potential Conflicts of Interest

The presence or absence of author conflicts of interest was determined by reviewing the authors' institutional affiliations, the conflicts of interest section, information on funding (if present) and the acknowledgements section.

The institutional affiliation of the first author (e.g., university, not-for-profit organization or for-profit agency) was retrieved from each article. The affiliation of the other authors was also recorded when a financial tie with a for-profit agency was present. Subsequently, the studies were classified for analysis as follows: (1) authors declaring no competing interests; (2) financial ties with the sponsor of the study disclosed, indicating that at least one author participated on behalf of a for-profit agency (e.g., as an employee or consultant); (3) other financial ties disclosed, indicating that an author did not participate on behalf of the study sponsor but did report a conflict of interest such as a past financial tie with the pharmaceutical industry; and (4) conflicts of interest not reported.

### Study Findings

For each published trial, the results for each primary outcome were classified as one of the following: (1) favourable, if the result was statistically significant with *P *< 0.05 or if the confidence interval did not include the null; or (2) inconclusive, if the result did not reach statistical significance.

For equivalence or noninferiority studies, the result was coded as favourable when the assumption of equivalence or noninferiority was satisfied.

### Statistical Methods

The distribution of various characteristics of the examined trials such as the type of ITT analysis and the source of funding were reported using descriptive statistics. The Pearson chi-square test was used for contingency table analysis. Interobserver agreement was measured using the kappa (κ) statistic.

We investigated the associations between the "type" of intention-to-treat analysis (as a dependent, multinomial 3-level variable: ITT, mITT, or "no ITT") and the methodological quality (sequence generation, allocation concealment and blinding; Model A), the quality of reporting (reporting of a flow-chart, sample size calculation, and lost to follow-up; Model A), the funding source (Model B), and the presence of authors' conflicts of interest (Model C) using multinomial logistic regression.

Bivariate analyses were performed to identify independent variables with a *P *value less than or equal to 0.10. These potential confounding variables were included in each model of multivariate multinomial logistic regression analysis. In each model, the variables of journal type, use of placebo, the presence of post-randomisation exclusions, and all of the items used as indicators of methodological quality and quality of reporting were included. In model A, in addition to all the mentioned variables, the use of funding was included (the inclusion of the presence of authors' conflict of interest instead of funding did not produce any change in the results of the model).

In each model, the ITT category was used as a reference for comparisons.

Furthermore, we performed univariate logistic regression to investigate the association between findings (as a dependent variable) and the "type" of intention-to-treat (as an independent polynomial 3-level variable: ITT, mITT, "no ITT"). To account for potential confounding variables we performed a multivariate logistic regression and included in the model the type of the journal, the use of placebo, the presence of post-randomisation exclusions, the use of funding and all the items that were indicators of reporting and methodological quality.

Finally, we assessed the overall association between favourable findings and no for-profit sponsorship using a logistic regression (univariate and multivariate model were used; all of the above mentioned variables including the "type" of intention-to-treat were used for adjustment). This was done to evaluate any consistency with studies in the biomedical literature that report a strong association between industry sponsorship and positive findings. The Pearson goodness-of-fit test was used to assess the overall fit of the models. Odds ratios (ORs) were then computed with confidence intervals (CIs).

A two-tailed *P*-value less than 0.05 was considered statistically significant. The analyses were performed using STATA/SE, version 8.2 for Windows (StataCorp, College Station, Texas).

## Results

### Characteristics of the Included Studies

Our final sample consisted of 367 published RCTs. Of these, 197 were classified as ITT trials, 114 as "no ITT" trials and 56 as mITT trials (analysis of number and types of mITT deviations are reported in Appendix 1).

The number of participants included in each RCT ranged from 10 to 160,921 (median, 368; interquartile range 140-991). Trials classified as mITT were significantly more likely to be published in general medical journals, to report post-randomisation exclusions and to use placebo as a comparator. A total of 258 (69%) trials received complete or partial financial support from a for-profit agency and 216 of the trials (60%) reported results that favoured the treatment under investigation. The characteristics of the included trials are shown in Table 1.

**Table 1:** Characteristics of Included Randomised Controlled Trials.

Characteristics	All trials	Type of Intention-to-treat reporting N (%)		
	**N = 367**	ITT 197 (54)	mITT 56 (15)	'no ITT' 114 (31)

**Journal**				

Medical journals	200 (55)	123 (62)	33 (59)	44 (38)

Specialty journals	167 (45)	74 (38)	23 (41)	70 (61)

**Study design features**				

Phase II	13 (4)	7 (4)	1 (2)	5 (4)

Equivalence/Noninferiority	31 (8)	14 (7)	9 (13)	10 (9)

Sample size [median, (IQR)]	368 (140-991)	476 (200-1160)	426 (198-1093)	201 (81-620)

Trials with post-randomization exclusions	177 (48)	84 (43)	37 (66)	56 (49)

Proportion of post-randomization exclusions [median, (IQR)]	4 (1-11)	3 (1-11)	4 (1-10)	5 (3-12)

Placebo use	123 (34)	51 (26)	27 (48)	45 (40)

**Reporting and Quality assessment**				

Flow chart	222 (60)	137 (70)	37 (66)	48 (42)

Reporting of sample size calculation	285 (78)	172 (87)	42 (79)	69 (61)

Reporting of lost to follow-up/withdrawal	211 (57)	127 (64)	35 (63)	49 (43)

Adequate sequence generation	167 (46)	108 (55)	21 (39)	38 (33)

Adequate allocation concealment	125 (34)	78 (40)	22 (39)	25 (22)

Adequate blinding	110 (30)	57 (29)	25 (45)	28 (25)

**Funding**				

Not-for-profit organization	137 (37)	88 (45)	9 (16)	40 (35)

For-profit agency	121 (33)	52 (26)	40 (71)	32 (28)

Co-financed	66 (18)	42 (21)	5 (9)	19 (17)

Not reported	43 (12)	18 (9)	2 (4)	23 (20)

**Author's conflict of interest**				

Author declaring no competing interests	113 (31)	74 (38)	8 (14)	31 (27)

Author on behalf of a for-profit agency	143 (39)	70 (36)	40 (71)	33 (29)

Author disclosing other financial ties	61 (17)	42 (21)	3 (5)	16 (15)

No report of competing interests	50 (14)	11 (6)	5 (9)	34 (30)

**Primary Outcome**				

Favorable to test drug	216 (60)	110 (56)	38 (70)	68 (60)

IQR = Inter-quartile range;

The kappa value for generation sequence was 0.79 (95% CI 0.74 to 0.85), for allocation concealment 0.82 (95% CI 0.77 to 0.88); for blinding, 0.78 (95% CI 0.72 to 0.84); and for intention-to treat analysis, 0.90 (95% CI 0.89 to 0.92). The kappa value for funding source was 0.96 (95% CI 0.95 to 0.97); for authors' conflict of interest, 0.94 (95% CI 0.93 to 0.96); and for study findings, 0.93 (95% CI 0.93 to 0.95).

### Characteristics of the Included Trials and the Type of Intention-to-treat Reporting

Multinomial logistic regression analyses showed that the mITT trials were more likely to have inadequate or unclear sequence generation and adequate blinding compared to the ITT trials. Trials classified as "no ITT" reported low standards of reporting and methodological quality.

In the multivariate analysis, the mITT trials appeared to have substantially similar methodological quality standards compared to the ITT trials; however, the mITT trials remained more likely to report post-randomisation exclusions (adjusted OR 3.43 [95% CI 1.70 to 6.95]; *P *= 0.001). Trials classified as "no ITT" were significantly more likely to avoid reporting flow-chart, sample size calculation and lost to follow-up. Table 2 shows the unadjusted and adjusted ORs of the strength of association between characteristics of the studies and the type of intention-to-treat reporting.

**Table 2:** Association Between Characteristics of the Included Randomised Controlled Trials and the Type of Intention-to-treat Reporting

Type of Intention-to-treat	Journal *Medical vs. specialty*	Placebo *Non-placebo vs. placebo*	Post-randomization exclusion *Reported vs. not reported*	Flow chart *Not reported vs. reported*	Sample size calculation *Not reported vs. reported*	Lost to follow-up *Not reported vs. reported*	Sequence Generation *Not adequate vs. adequate*	Allocation concealment *Not adequate vs. adequate*	Blinding *Not adequate vs. adequate*
	**Unadjusted Odds Ratio (Confidence Interval 95%)**								

**mITT**	1.16 (0.63 to 2.12)	2.67 (1.44 to 4.92)	2.62 (1.41 to 4.88)	1.17 (0.62 to 2.20)	1.87 (0.87 to 4.02)	1.09 (0.59 to 2.01)	1.42 (1.05 to 1.93)	1.01 (0.74 to 1.36)	0.50 (0.27 to 0.93)

**"no ITT"**	2.64 (1.64 to 4.25)	1.87 (1.14 to 3.05)	1.30 (0.82 to 2.06)	3.14 (1.94 to 5.07)	4.47 (2.55 to 7.88)	2.41 (1.50 to 3.86)	1.52 (1.23 to 1.98)	1.53 (1.17 to 1.99)	1.25 (0.74 to 2.12)

	**Adjusted Odds Ratio (Confidence Interval 95%)**								

**mITT**	1.43 (0.61 to 3.35)	2.85 (1.22 to 6.63)	3.43 (1.70 to 6.95)	1.05 (0.47 to 2.37)	3.24 (1.32 to 7.98)	1.13 (0.54 to 2.36)	1.15 (0.79 to 1.69)	0.98 (0.69 to 1.41)	1.00 (0.43 to 2.34)

**"no ITT"**	1.87 (0.94 to 3.69)	4.11 (2.02 to 8.36)	2.27 (1.30 to 3.93)	1.92 (1.03 to 3.55)	4.05 (2.10 to 7.84)	2.09 (1.20 to 3.66)	1.06 (0.79 to 1.43)	1.24 (0.91 to 1.69)	1.88 (0.90 to 3.94)

*ITT is the comparison group*.

mITT = modified intention-to-treat

ITT = intention-to-treat.

### Sponsorship, Conflicts of Interest and the Type of Intention-to-treat Reporting

Compared to the ITT trials, RCTs classified as mITT were more likely to receive sponsorship from a for-profit agency (adjusted OR 7.41 [95% CI 3.14 to 17.48]; *P *< 0.001) and were more likely to have at least one investigator who was authoring on behalf of the pharmaceutical industry (adjusted OR 5.14 [95%CI, 2.12 to 12.48]; *P *< 0.001). Interestingly, both the mITT and "no ITT" trials reported a higher odds ratio of authors not reporting any conflicts of interest. Table 3 shows the unadjusted and adjusted ORs of the strength of association between funding and authors' conflicts of interest and the type of intention-to-treat reporting.

**Table 3:** Association Between Funding and Authors' Conflicts of Interest for the Included Randomised Controlled Trials and the Type of Intention-to-treat Reporting.

Type of Intention-to-treat	Funding				Authors' Conflicts of Interest			
	**Not for-profit organization**	**For-Profit Agency**	**Co-financed**	**Not Reported**	**Author declaring no competing interests**	**Author on behalf of a for-profit agency**	**Author disclosing other financial ties**	**No report of competing interests**

	**Unadjusted Odds Ratio (Confidence Interval 95%)**							

**mITT**	1	7.99 (3.59 to 17.82)	1.16 (0.37 to 3.69)	1.09 (0.22 to 5.46)	1	5.29 (2.31 to 12.08)	0.66 (0.16 to 2.63)	4.20 (1.16 to 15.19)

**"no ITT"**	1	1.44	0.99	2.81	1	1.12	0.91	7.38
		(0.80 to 2.57)	(0.52 to 1.92)	(1.37 to 5.78)		(0.62 to 2.03)	(0.45 to 1.85)	(3.32 to 16.40)

	**Adjusted Odds Ratio (Confidence Interval 95%)**							

**mITT**	1	7.41 (3.14 to 17.48)	1.08 (0.32 to 3.58)	0.87 (0.16 to 4.71)	1	5.14 (2.12 to 12.48)	0.62 (0.15 to 2.61)	4.78 (1.03 to 22.08)

**"no ITT"**	1	1.23 (0.62 to 2.43)	0.82 (0.39 to 1.72)	1.31 (0.54 to 3.15)	1	0.88 (0.45 to 1.73)	0.60 (0.26 to 1.38)	3.77 (1.30 to 10.94)

ITT is the comparison group.

mITT = modified intention-to-treat

ITT = intention-to-treat.

### Analysis of the Findings

We did not find any association between mITT reporting and favourable results (adjusted OR 1.27 [95% CI 0.62 to 2.61]; *P *= 0.51). Overall, however, trials with for-profit agency sponsorship were more likely to report favourable results when compared to trials that did not receive any sponsorship from a for-profit agency (adjusted OR 2.30 [95% CI 1.28 to 4.16]; *P *= 0.006).

## Discussion

In a sample of RCTs, we assessed the associations between the type of intention-to-treat analysis used and study design characteristics, including the source of funding and the favourability of the results. Our first hypothesis that studies using mITT analyses would be associated with limited quality of reporting and study characteristics was refuted. Instead, we found that the mITT trials were methodologically similar to the ITT trials.

Reports from the literature about the methodological quality of industry sponsored trials are controversial. While some authors report that the methodological quality of industry-sponsored RCTs is limited, others report that trials funded by for-profit agencies have similar or better methodological quality than unsupported trials [8,11].

In our investigation, the mITT trials were more likely to perform post-randomisation exclusions compared to both ITT trials and "no ITT" trials. A study published in 1996 reported a paradox that RCTs reporting ITT with sound methodological quality were more likely to perform exclusions [12]. The authors' interpretation of this finding was that studies with low methodological standards may be less likely to report exclusions, even when exclusions actually occurred. In our analysis, although the mITT trials had the highest occurrence of exclusions, we found that the "no ITT" trials were more likely to perform exclusions compared to the ITT trials. It is possible that the standard of reporting changed over time, and we can speculate that allowing any modification of the standard intention-to-treat analysis may have encouraged authors to report exclusions after deciding which "type" of mITT analysis to perform.

Several studies in the medical literature report that post-randomisation exclusions in randomised trials may lead to biased estimates of the treatment effect. Individual patient data analyses of systematic reviews found that the results were in favor to the treatment under investigation when exclusions were not taken into account in the results rather than when a true intention-to-treat was used[13]. A meta-epidemiological study that investigated 14 meta-analyses in osteoarthritis showed that the exclusion of patients from analysis resulted in a biased estimate of the treatment effect [14]. Another study of pharmaceutical industry sponsored trials investigating serotonin reuptake inhibitors found that results were more in favour when a per-protocol analysis was used instead of intention-to-treat analysis [15].

The second aim of our study was to assess the associations between the type of intention-to-treat analysis used and the presence of industry sponsorship or author conflicts of interest among RCTs. In the RCTs included in this study the use of mITT analysis was strongly associated with industry funding and author conflicts of interest.

In the medical literature, there is plenty of evidence that the pharmaceutical industry is directly or indirectly involved at different stages in the conduct, design and publication of biomedical research[9,11,16]. The selection of an inappropriate comparison group (e.g., a drug with non-equivalent dosage) [8,17,18], multiple reporting of studies [19,20] and suppression or delay of publications [18] are all circumstances where the presence of industry sponsorship are well documented.

We did not find any association between favourable results and mITT reporting. The number of mITT trials was too low to detect any difference; however, even if the sample of trials was adequate, we are aware that any potential difference cannot be free of bias given the large heterogeneity of the included trials in terms of the intervention and outcome investigated. We believe, nonetheless, that the appropriate place to evaluate the mITT as a source of bias is to explore its impact in meta-epidemiological studies.

In our previous survey, we showed that publications of mITT reporting trials is significantly increasing. For example, the overall incidence of trials published in 2006 in the medical literature was around 5%[5]. This incidence of mITT reporting trials was underestimated since the studies claiming the use of the intention-to-treat approach when in fact they used the mITT approach (owing to the type of deviation present in the description of the analysis) may have escaped the previous search[5]. Indeed, in the present study, we were able to assess all the randomized trials in 6 journals, and capture those trials that reported intention-to-treat but with deviations. The number of these trials (n = 32) was greater than the number of the trials that actually reported as "modified" (n = 24; Box). Consequently, we can conclude that the phenomenon of mITT is wider than previously estimated.

## Limitations

Our main limitation was that we used data from RCTs that were published in six journals; therefore, we are unsure that our results can be generalized to other trials published elsewhere. To test our aforementioned hypothesis, we needed to compare mITT trials with trials reporting (or not reporting) intention-to-treat. Although our previous research has documented an increasing incidence of mITT trials, the proportion of mITT trials being conducted remains too low to obtain an adequate sample for comparison. Consequently, we decided to target journals that were more likely to report mITT trials by selecting general medical and specialty journals.

## Conclusion

Data analysis is the crucial final stage of any study design. Well-designed RCTs require strict adherence to the intention-to-treat principle, in which subjects should remain in the group to which they were originally allocated. Although further research is needed to document that the mITT is a potential source of bias, to limit unjustified exclusions, the misuse of "intention-to-treat" terminology should be abandoned, and authors need to adhere to the standard intention-to-treat principle. The updated version of the CONSORT statement goes into this direction and suggests the replacement of any kind of intention-to-treat reporting with a clear description of exactly who was included in each analysis[21].

## Appendix 1

Analyses of Types and Deviations from Intention-to-Treat of Trials classified as mITT

Of the 56 trials classified as mITT, 24 were reported explicitly as "modified" while 32 reported "intention-to-treat" but reported descriptions that deviate from the true ITT and thus were considered as mITT trials.

Overall, 31 (55%) trials reported 1 type of mITT deviation, 17 (30%) reported 2 types of mITT deviation, 5 (9%) reported 3 types of mITT deviation, and 3 (5%) did not report any type of mITT deviation. In 35 (63%) trials, the main exclusion criterion for the mITT analysis was treatment-related; in 18 of such trials, the treatment-related mITT was accompanied by at least one additional type of mITT deviation.

In 17 (30%) trials, the exclusion criteria used to justify the mITT criteria was the absence of a post-baseline assessment; in 13 of these trials, it was accompanied by at least one other type of mITT deviation.

Baseline assessment related mITT was described in 10 (18%) trials; in 6 of these the approach was accompanied by another type of deviation. Four (7%) trials required a target condition to describe the mITT deviation; and 1 (2%) trial fell into the follow-up related mITT deviation. While 13 (23%) trials did not fall into none of the above category, 3 (5%) trials remained without any type of mITT description.

## Competing interests

Maria Laura Luchetta has been employed in Laboratori Guidotti s.p.a. (Pisa) from November 2003 to October 2006; in Menarini IFR (Firenze) from November 2006 to November 2007. Alessandro Montedori, Maria Isabella Bonacini, Giovanni Casazza, Francesco Cozzolino, Piergiorio Duca, and Iosief Abraha declare that they have no competing interests.

## Authors' contributions

AM and IA conceived and designed the study. AM, MIB, FC, MLL collected data. GC, PD, IA did the statistical analyses. All authors contributed to the interpretation of the results, the writing and critical review of the report. All authors have seen and approved the final version of the report.
